# Agar-tragacanth/silk fibroin hydrogel containing Zn-based MOF as a novel nanobiocomposite with biological activity

**DOI:** 10.1038/s41598-024-61329-3

**Published:** 2024-05-07

**Authors:** Farnaz Lalebeigi, Amir Kashtiaray, Hooman Aghamirza Moghim Aliabadi, Fatemeh Moghadaskhou, Zeinab Pajoum, Seyede Mehrnoush Nokandeh, Mohammad Mahdavi, Reza Eivazzadeh-Keihan, Ali Maleki

**Affiliations:** 1 Catalysts and Organic Synthesis Research Laboratory, Department of Chemistry, Iran University of Science and Technology, Tehran, 16846-13114 Iran; 2 Medical Biotechnology Department, Biotechnology Research Center (BRC), Pasteur Institute of Iran (IPI), Tehran, Iran; 3 Endocrinology and Metabolism Research Center, Endocrinology and Metabolism Clinical Sciences Institute, Tehran University of Medical Sciences, Tehran, Iran

**Keywords:** Agar, Tragacanth gum, Silk fibroin, MOF-5, Biological activity, Biochemistry, Carbohydrates

## Abstract

In this study, a novel nanobiocomposite consisting of agar (Ag), tragacanth gum (TG), silk fibroin (SF), and MOF-5 was synthesized and extensively investigated by various analytical techniques and basic biological assays for potential biomedical applications. The performed Trypan blue dye exclusion assay indicated that the proliferation percentage of HEK293T cells was 71.19%, while the proliferation of cancer cells (K-562 and MCF-7) was significantly lower, at 10.74% and 3.33%. Furthermore, the Ag-TG hydrogel/SF/MOF-5 nanobiocomposite exhibited significant antimicrobial activity against both *E. coli* and *S. aureus* strains, with growth inhibition rates of 76.08% and 69.19% respectively. Additionally, the hemolytic index of fabricated nanobiocomposite was found approximately 19%. These findings suggest that the nanobiocomposite exhibits significant potential for application in cancer therapy and wound healing.

## Introduction

Hydrogels are cross-linked network matrices composed of various polymers, which possess hydrophilicity, plasticity, super absorbency, and softness features. The unique physicochemical properties of hydrogels have been considered for use in widespread fields such as adsorption^1^, coating^2^, cancer therapy^3^, producing contact lenses^4^, wound dressing^5^, drug delivery^6^, and tissue engineering^7^. Hydrogels are categorized into natural, synthetic, and hybrid gels based on their constituent polymers. Natural hydrogels are good candidates for biomedical applications due to their low cytotoxicity, biodegradability, and biocompatibility properties^8–10^. The natural hydrogels based on chitosan, alginate, gelatin, and hyaluronic acid exhibit antibacterial properties by disrupting bacterial cell membranes and interfering with protein synthesis and DNA replication^11^. Additionally, these hydrogels can mimic the natural extracellular matrix^12^, facilitating cell adhesion^13^, proliferation, and interaction through cell–matrix interactions. The porous structure of these hydrogels enables the diffusion of nutrients, promoting cell viability and function^14^. Ag is a well-known natural polymer widely used as a linear polymer in hydrogel networks. Ag is a thermosensitive and cost-effective natural polysaccharide obtained from seaweed (red algae). It is commonly used in gel electrophoresis to separate DNA^15^. The presence of abundant hydroxyl functional groups in Ag provides active sites for interaction with monomers and cross-linkers, thereby enabling the formation of the hydrogel network structure. TG is another polysaccharide polymer with a natural origin that has been used as a stabilizing, emulsifying, and thickening agent in the food and pharmaceutical industries^16^. TG, consisting of highly branched chains, is a dried exudate extracted from the *Astragalus gummifer* plant^17^. Like Ag, TG possesses many hydroxyl functional groups in their respective structures. These functional groups are crucial in facilitating the formation of multi-polymeric hydrogels. Recently, several studies have been conducted on multi-polymeric hydrogels, such as sodium alginate-chitosan^18^, pectin-cellulose^19^, lignin-xanthan^20^, gelatin-starch^21^, etc. The preparation of these hydrogels involves the use of various organic^22^ and inorganic^23^ cross-linkers. These cross-linkers facilitate the formation of covalent and non-covalent interactions between different functional groups of the polymers. In this research, glutaraldehyde, an organic cross-linker, was used to form the Ag-TG hydrogel. Glutaraldehyde links two desired polymer chains through its two aldehyde functional groups^24^. This cross-linking process results in the development of a three-dimensional hydrogel structure with interconnected pores, enhancing its stability and porosity. Another advantage of these hydrogels is their ability to incorporate with other biological macromolecules, such as nucleic acids and proteins, which will improve their biomedical applications. In recent years, SF has gained significant attention among researchers as a natural protein with versatile properties. It can be combined with various components, making it an attractive material for a wide range of applications^25^. SF is derived from the *Bombyx mori*, commonly known as the silkworm. SF predominantly comprises two principal proteins, fibroin and sericin^26^. The adhesive sericin is a twisted protein around fibroin, and it can be isolated from the SF by different methods. Biocompatibility, non-cytotoxicity, antibacterial activities, high mechanical performance, and sufficient production provide the usage of SF in vast fields, such as drug delivery, gene therapy, wound healing, and bone and vascular tissue regeneration^27–29^. All natural biomacromolecules used in Ag-TG hydrogel/SF composite are biodegradable and can be broken down by enzymes or microorganisms^30^. Biodegradable hydrogels are designed to degrade over time, either through enzymatic or hydrolytic processes, allowing for the controlled release of drugs within the body^31^. One crucial biological characteristic that distinguishes composites from other materials is their ability to exhibit an antibacterial activity, which can be induced by utilizing various metal–organic frameworks (MOFs)^32^. MOFs are highly porous hybrid materials consisting of inorganic metal ions and organic ligands. These structures have garnered considerable attention due to their versatility and wide applications in the field of biotechnology^33^. Previous studies have displayed that MOFs operate as third-generation antibacterial agents through controlled ion release and interaction with bacteria cell walls^34^; thus, combining MOF-5 with Ag-TG/SF hydrogel enhances its antibacterial properties. MOF-5, also known as IRMOF-1, is a three-dimensional cubic porous framework with the chemical formula [Zn_4_O(BDC)_3_]. Its exceptional surface area makes it highly suitable for various applications, including biosensing, gas storage, and drug release^35–37^.

In this study, a novel nanobiocomposite based on Ag, TG, glutaraldehyde, SF, and MOF-5 was prepared in a facial approach, then its proliferation, lytic effect on cells, and the antibacterial activity were investigated by trypan blue exclusion, hemolysis, and antibacterial assays. The proliferation percentage of HEK293T, MCF-7, and K-562 cells indicated that the Ag-TG hydrogel/SF/MOF-5 nanobiocomposite exhibited an anticancer effect against cancer cells. In addition, a significant decrease in bacterial growth was observed for both E. coli and S. aureus strains, with inhibition rates of 76.08% and 69.19% respectively. The results demonstrate that this novel nanobiocomposite is an appropriate candidate for wound healing and cancer therapy applications.

## Experimental

### Materials

All materials used in the synthesis of nanobiocomposites and biological assays, with the exception of silkworm cocoons, were obtained from reputable Sigma-Aldrich company. These materials include agar (Ag), tragacanth gum (TG), glutaraldehyde (25% in H_2_O), silk cocoon (*Bombyx mori*) (3 silk cocoons), HCl (37 wt.%), Na_2_CO_3_, LiBr, trimethylamine, terephthalic acid, Zn(OAC)_2_⋅2H_2_O, dimethylformamide (DMF), CHCl_3_, Phosphate buffer saline (PBS) (pH  7.4 for cell culture), Trypan blue dye, Bovine serum (FBS), Triton X-100, NaCl (0.9%), Mueller Hinton Broth (MHB), Mueller Hinton agar (MHA), RPMIv1640 medium, and DMEM/F12 medium supplements. Additionally, a dialysis tubing cellulose membrane with a molecular weight of 14 kilodaltons was sourced from Sigma-Aldrich. Silk cocoons were obtained from local producers, specifically Farasahel Negar Company.

### Instruments

To characterize the physicochemical properties of the nanobiocomposite, various devices such as Fourier-transform infrared (FT-IR) spectrometer (PERKIN ELMER spectrum 100), X-ray diffractometer (Bruker, D8 advance), thermogravimetric analyzer (TGA 2-METTLER TOLEDO), energy-dispersive X-ray (EDX) spectrometer (oxford instruments), field-emission scanning microscope (FE-SEM) (SIGMA VP- ZEISS) were applied. In addition, an ELISA Reader (Biohit, Finland) was employed for the biological assays.

### Preparation of Ag-TG hydrogel

**Figure 1:**
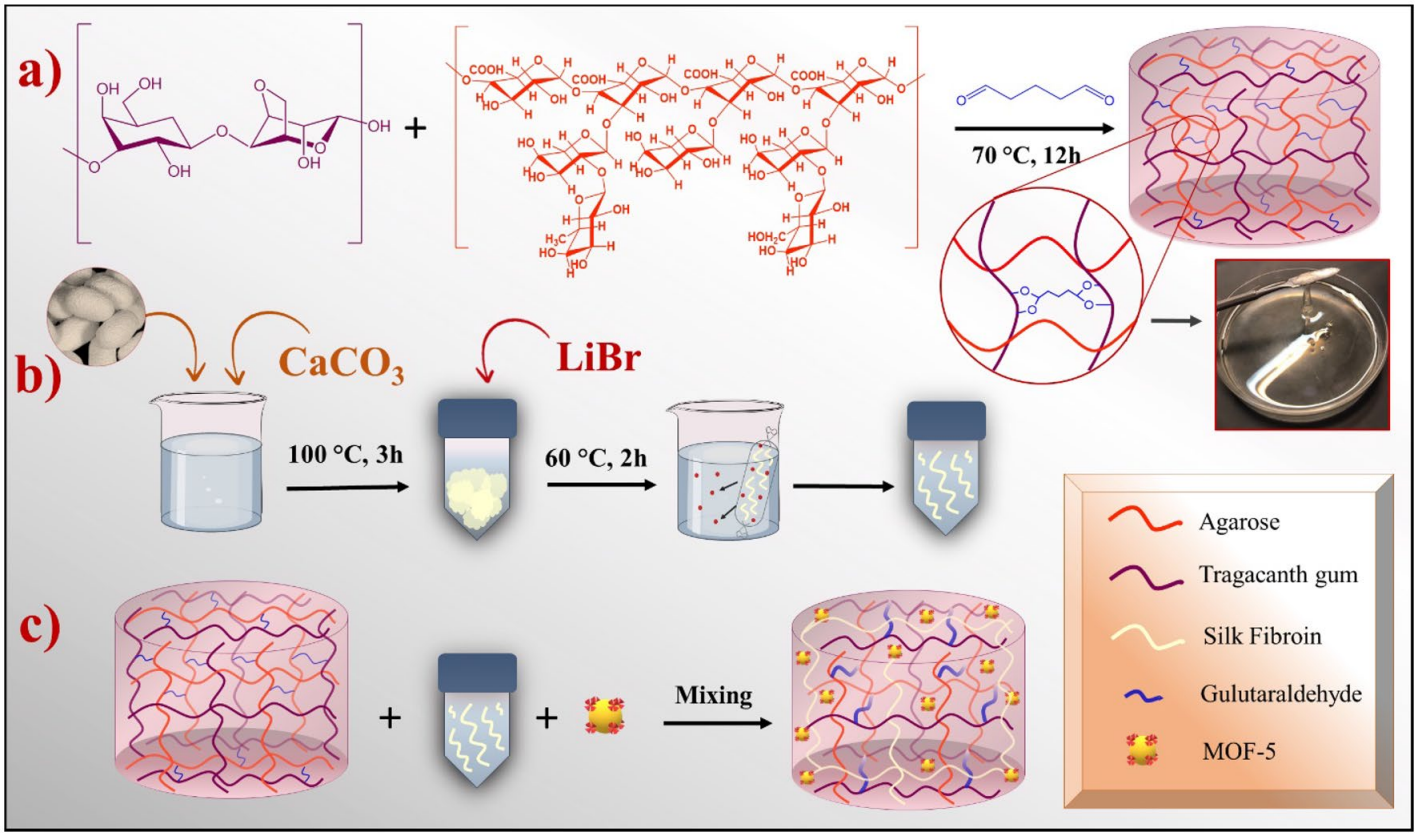
Schematic illustration of Ag-TG hydrogel preparation (**a**), extraction of SF (**b**), and synthesis of Ag-TG hydrogel/SF/MOF-5 nanobiocomposite.

Initially, the experimental procedure involved the dissolution of Ag (0.5g) and TG (0.5g) in 50 mL of distilled water individually. Subsequently, two drops of an HCl solution (37 wt.%) were added to the resultant mixture. Afterward, the entirety of the prepared solution was stirred for 30 min at 70 °C until effective homogenization of the respective polymers was achieved. In the experimental method shown in Fig. 1a, 2 mL of glutaraldehyde was added dropwise to the final solution with a time interval of 10 min. The solution was then kept under stirring conditions for 12 h at 70 °C.

### Extraction of SF

According to the previous studies^38^, at first, three pieces of silkworm cocoons were boiled in 500 mL of aqueous Na_2_CO_3_ solution (0.025 M) for 2 h until the yellow sericin protein was separated; then, the degummed white cocoons were cleanly washed three times with distilled water and were dried in ambient conditions. After completely drying fibroin protein fibers, they were weighed (0.583 g) and then dissolved in LiBr aqueous solution with a concentration of 9.3 M. This dissolution process was carried out for 2 h at 60 °C. The solution was dialyzed by an appropriate dialysis cellulose membrane for three days with frequent exchange of distilled water. This dialysis process effectively removed LiBr from the solution during the rehydration process. After the mentioned time, the extracted SF was provided for the next steps (Fig. 1b).

### Preparation of Ag-TG hydrogel/SF

In this step, the Ag-TG hydrogel/SF was prepared by combining 2.5 mL of Ag-TG hydrogel (1.89% w/v) and 2.5 mL of extracted SF (1.8% w/v). The mixture was continuously stirred for 3 h. After the mentioned time, the solution was carefully transferred into a Petri dish and set aside for the final addition of MOF-5.

### Synthesis of MOF-5

In the first step, a solution was prepared by dissolving 8.5 mL of triethylamine and 5.065 g of terephthalic acid (30.5 mmol) in 400 mL of DMF. In a separate step, another solution was prepared by dissolving 16.99 g of Zn(OAC)_2_⋅2H_2_O (77.4 mmol) in 500 mL of DMF. The second solution was then added to the primary organic solution while stirring for 15 min to produce a precipitate. The resulting solution was further stirred for 150 min. After mentioned time, the residue was filtered and soaked in 250 mL of DMF for 24 h. The solution underwent secondary filtration and was transferred to a container with 350 mL of CHCl_3_. The solvent used in the process was changed three times over 1 week. The first change occurred after two days, the second after 3 days, and the third after 7 days. Finally, the ultimate product was activated by heating it at 120 °C for 6 h^39^.

### Preparation of Ag-TG hydrogel/SF/MOF-5

To prepare the final nanobiocomposite, 0.02 g of MOF-5 was accurately measured and dispersed in 2 mL of distilled water. The resulting dispersion was added to 5 mL of the Ag-TG hydrogel/SF composite. This mixture was stirred for 3 h. After the mentioned time, the synthesized nanobiocomposite was poured into a Petri dish for freeze-drying and dehydration at -60°C for 24 h. After freeze-drying, the final dried nanobiocomposite is ready for biological investigation and determination of its physiochemical properties (Fig. 1c).

### Nanobiocomposite extraction for biological assays

The extraction process involved dissolving 50 mg of the Ag-TG/SF/MOF-5 nanobiocomposite in 1 ml of PBS. The dissolution was carried out in a shaker incubator at 37 °C for 48 h^40,41^.

### Trypan blue exclusion test

To investigate the biocompatibility and anticancer activity of the Ag-TG/SF/MOF-5 nanobiocomposite, three cell lines were used: HEK293T (human embryonic kidney cell line), MCF-7 (human breast cancer cell line), and K-562 (human erythroleukemia cancer cell line). These cell lines were obtained from the Pasteur Institute of Iran.

The HEK293T and MCF-7 cell lines were cultured in DMEM/F12 medium supplemented with 10% fetal bovine serum. On the other hand, the K-562 cell line was cultured in RPMIv1640 medium also supplemented with 10% fetal bovine serum. After culturing, the cells were seeded at a density of 104 cells per well in a 24-well plate and incubated for 48 h. Next, 40 µl of the nanobiocomposite extract was added to the cells, and they were treated for 48 h. Each sample was tested in triplicate. The PBS-treated cell lines were considered as the negative control. Finally, the treated cells were collected and diluted tenfold with Trypan blue dye. The cell count was performed using a Neubauer chamber hemocytometer. The proliferation percentage of the treated cells was calculated using the following formula:^25,27,42^.

$$\mathrm{Proliferation} \%=\left(\frac{\mathrm{Average \, number  \, of \,  viable \,  cells \,  per \,  ml  \, in  \, treated \,  wells}}{\mathrm{Average  \, number \,  of \,  viable  \, cells \,  per  \, ml  \, in  \, control \,  wells}}\right) \,  \times  \, 100$$

### Hemolysis assay

To investigate the blood compatibility of the Ag-TG/SF/MOF-5 nanobiocomposite, the percentage of human red blood cell (RBC) lysis was measured. Firstly, a fresh O-negative blood group sample was obtained from a volunteer and diluted in PBS at a ratio of 1:20. Then, 100 µl of the diluted blood sample were mixed with 100 µl of the nanobiocomposite extract in a 96-well plate. The plate was then incubated at 37 °C for 1 h. After incubation, the samples were collected and centrifuged at 3000 rpm for 15 min. In this assay, 1% Triton X-100 was used as the positive control, while 0.9% NaCl solution was used as the negative control. Supernatant photometric analysis was performed to quantify the absorbance values at a wavelength of 405 nm using an ELISA Reader. The hemolysis percentage of the samples was determined utilizing the following formula^43^:

$$\mathrm{Hemolysis}\,\%= \left[\frac{\mathrm{mean  \, OD  \, of \,  sample}-\mathrm{mean  \, OD  \, of  \, negative  \, control}}{\mathrm{mean \,  OD \,  of  \, positive \,  control}-\mathrm{ mean \,  OD  \, of  \, negative \,  control}}\right] \,  \times \,  100$$

### Antibacterial assay

To assess the antibacterial properties of the Ag-TG/SF/MOF-5 nanobiocomposite, the inhibition rate of *E. coli* and *S. aureus* bacteria was measured^44,45^. For this purpose, E. *coli* and S. *aureus* bacteria were cultured overnight in an MHA culture medium. Afterward, the bacteria were inoculated into an MHB culture medium and incubated until they reached a final McFarland concentration 0.5. Next, 50 μL of each inoculated bacteria and 0.01 g of the Ag-TG/SF/MOF-5 nanobiocomposite were added to two test tubes containing 5 ml of sterile MHB culture medium. These test tubes were then placed in a shaker incubator set at 37 °C to provide optimal growth conditions for the bacteria. Agitation was achieved by setting the shaker incubator at 150 rpm for 24 h. All experiments were repeated three times. Finally, the absorbance of each tube was measured at a wavelength of 600 nm; then, the growth inhibitory percentage was calculated using the following mathematical expression:

$$\mathrm{Growth\,inhibition} \%=\left(1-\frac{\mathrm{Mean \, OD \, of \, sample}}{\mathrm{Mean \, OD\,  of\,  positive\,  control}}\right)\, \times \, 100$$

### Statistical analysis

The statistical software SPSS Statistics 22.0 was used to perform data analyses using the t-test, an inferential technique. In statistical analysis, P-values of P ≥ 0.05 (*), P ≤ 0.05 (**), and P ≤ 0.001 (***) corresponded to statistical insignificance, significance, and high significance, respectively. The error bar used in this study was of the standard deviation type.

### Ethical issues

The present investigation complied with the ethical principles stipulated in the Declaration of Helsinki. Furthermore, it should be noted that the Ethics Research Committee of the Pasteur Institute of Iran sanctioned the investigative techniques employed and the protocol for obtaining informed consent.

## Result and discussion

In this study, a novel nanobiocomposite with biocompatible, non-toxic, anti-cancer and antibacterial properties was developed through a three-step process. The first step involved synthesizing a cross-linked multi-polymeric Ag-TG hydrogel using glutaraldehyde as a cross-linker. In the second step, SF and MOF-5 were incorporated into the hydrogel to enhance its biological and antibacterial properties. Various analytical techniques, such as FT-IR, EDX, FE-SEM, XRD, and TGA, were performed to determine the functional group characterization, elemental composition, morphology, crystallinity, and thermal stability of the nanobiocomposite.

### FT-IR analysis

**Figure 2:**
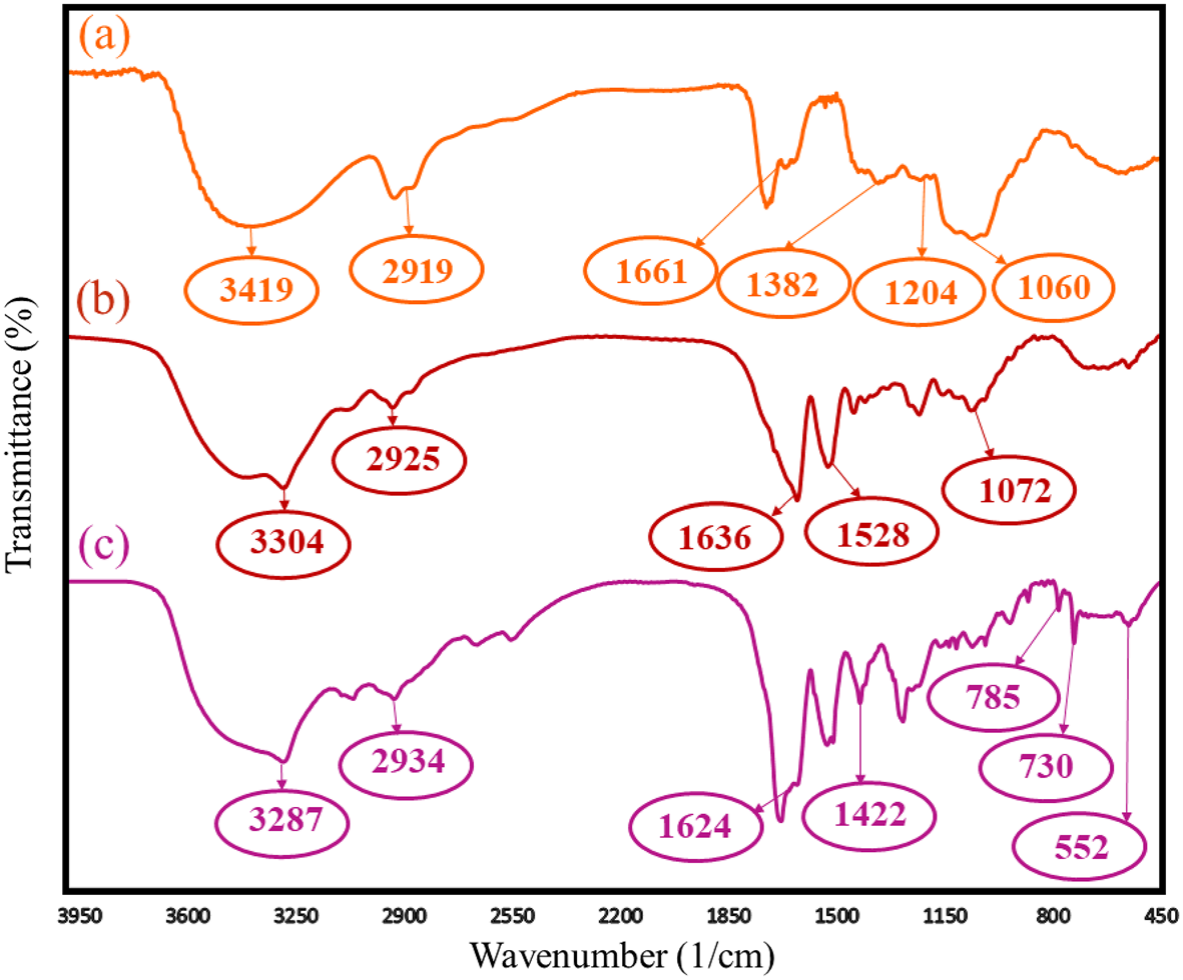
FT-IR spectra of Ag-TG hydrogel (**a**), Ag-TG hydrogel/SF (**b**), Ag-TG hydrogel/SF/MOF-5 (**c**).

FT-IR analysis is a widely used technique for determining the functional groups and chemical bonds of synthesized composites. In this research, FT-IR analysis was applied to investigate the structure of the nanobiocomposite components at each step of preparation. All FT-IR spectra of Ag-TG hydrogel, Ag-TG hydrogel/SF, and Ag-TG hydrogel/SF/MOF-5 have been exhibited in Fig. 1. In Fig. 2a, the strong, broad peak (3200–3600 cm^−1^) is related to the abundant O–H stretching vibration bands of Ag and TG polymers presented in the hydrogel structure^46^. The C-H stretching vibration was determined by absorption bands at 2919 cm^−1^. The assigned peaks at 1661 cm^−1^ and 1382 cm^−1^ are related to C=O and C–OH deformation vibrations respectively, as well as the O–C–O symmetric stretching vibration of carboxylate groups in TG^16^. The broad absorption peak at the range of (1000–1200 cm^−1^) has been identified as C–O (ether) and C–O–C (acetal ring) bands. This peak signifies the formation of cross-linking bonds within the hydrogel structure^47,48^. In Fig. 2b, additional peaks related explicitly to SF bonds were observed alongside the peaks corresponding to the Ag-TG hydrogel. The peak at 3304 cm^−1^ is attributed to the N–H stretching vibrations band, and the absorption peak at 1636 cm^−1^ is related to C=O bands of amide I in SF fibers. It is worth noting that the wavelength of this peak is lower than the carboxylic functional groups. Moreover, the assigned band at 1528 cm^−1^ is related to the N–H bending vibration mode of amide II. This particular band is ascribed explicitly to β-sheets in the structure of SF^49^. In Fig. 2c, after adding MOF-5 to the Ag-TG hydrogel/SF, several peaks were observed in the FT-IR spectrum specifically in the region under 1000 cm^−1^. The absorption band at 552 cm^−1^ is attributed to the Zn–O stretching band. The two close peaks at 728 cm^−1^ and 785 cm^−1^ are related to the in-plane bending of C–H bonds in the benzene ring in the MOF-5^50^. Also, the symmetric and asymmetric stretching of the carboxyl group in BDC (benzene-1,4-dicarboxylate), which is bonded to Zn, are obtained at two wavelengths: 1624 cm^−1^ and 1422 cm^−1^^51^.

### FE-SEM imaging

**Figure 3:**
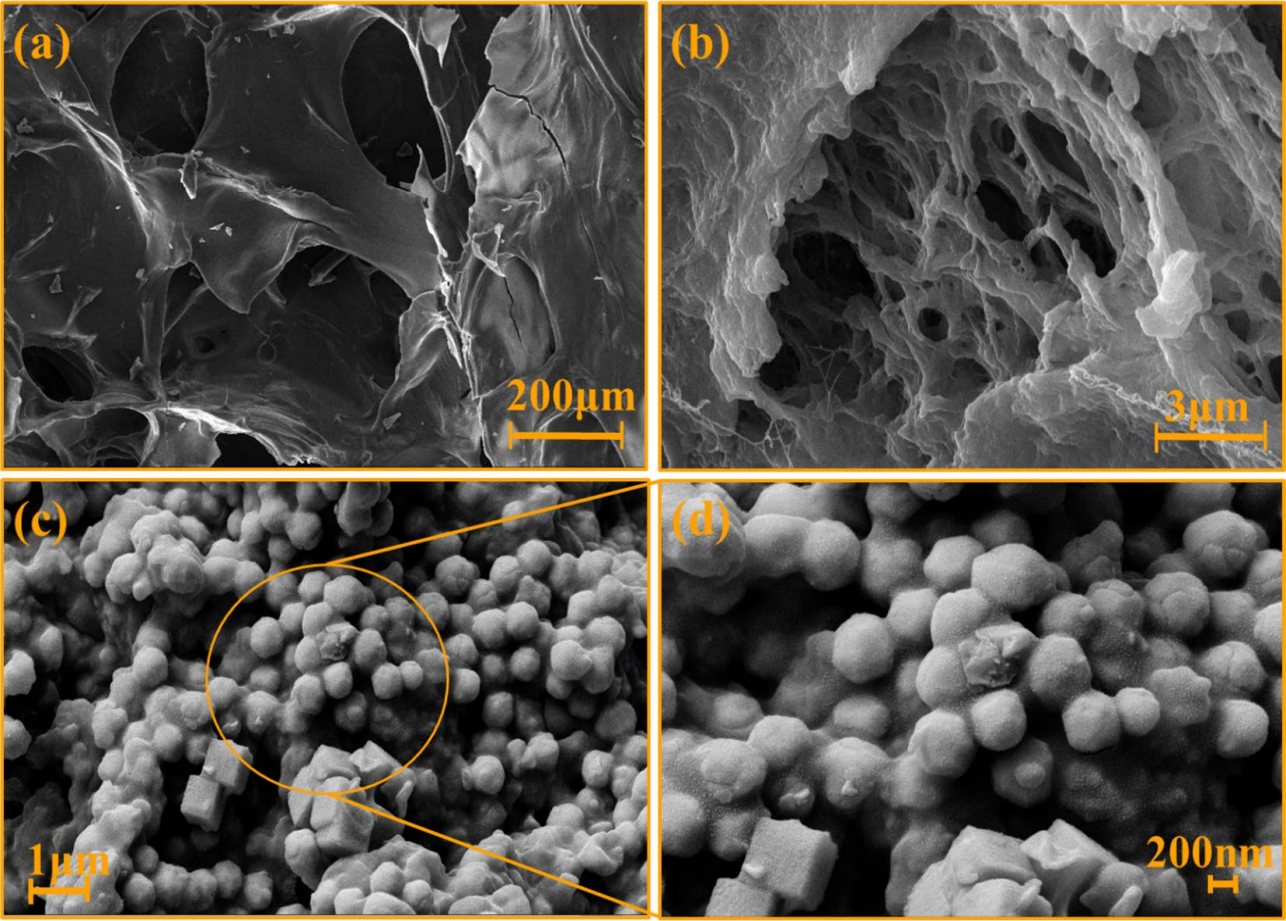
FE-SEM images of Ag-TG hydrogel (**a**), Ag-TG hydrogel/SF (**b**), Ag-TG hydrogel/SF/MOF-5 nanobiocomposite in different magnification (1 μm and 200 nm) (**c,d**).

For determination of the morphology of the nanobiocomposite components, FE-SEM images were taken after the preparation of all three samples: Ag-TG hydrogel, Ag-TG hydrogel/SF, and Ag-TG hydrogel/SF/MOF-5 (Fig. 3). Figure 3a, which is related to Ag-TG hydrogel, it can be observed that the hydrogel has a highly porous structure. This porous structure makes it a suitable substrate for cell growth and proliferation in wound healing applications. In Fig. 3b, the porosity of the hydrogel is maintained, but with the difference that the SF biopolymer strands cover the surface of the hydrogel. In Fig. 2c, with higher magnification, the MOF-5 was observed definitely in the final structure of the composite loaded in Ag-TG hydrogel/SF (Fig. 3c, d).

### EDX analysis

**Figure 4:**
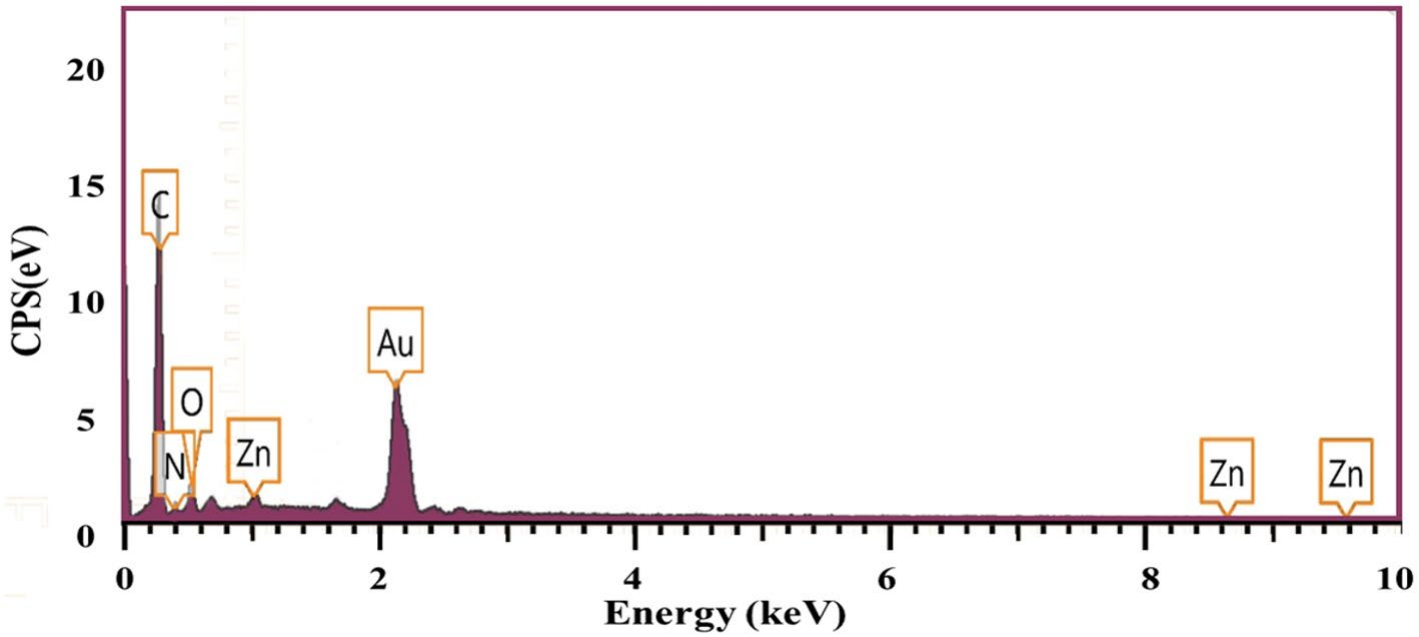
The EDX spectrum of Ag-TG hydrogel/SF/MOF-5 nanobiocomposite.

The EDX analysis was performed to specify the elemental structure of the synthesized Ag-TG hydrogel/SF/MOF-5 nanobiocomposite. The spectrum obtained from the EDX analysis exhibits that the C and O peaks corresponded to the used biopolymers, including Ag, TG, and SF, as well as the glutaraldehyde cross-linking agent. The N peak confirms the existence of SF which contains amide functional groups in its amino acid units. Also, the Zn peak is correlated with MOF-5, which is loaded in the final nanobiocomposite (Fig. 4).

### X-ray diffraction analysis

**Figure 5:**
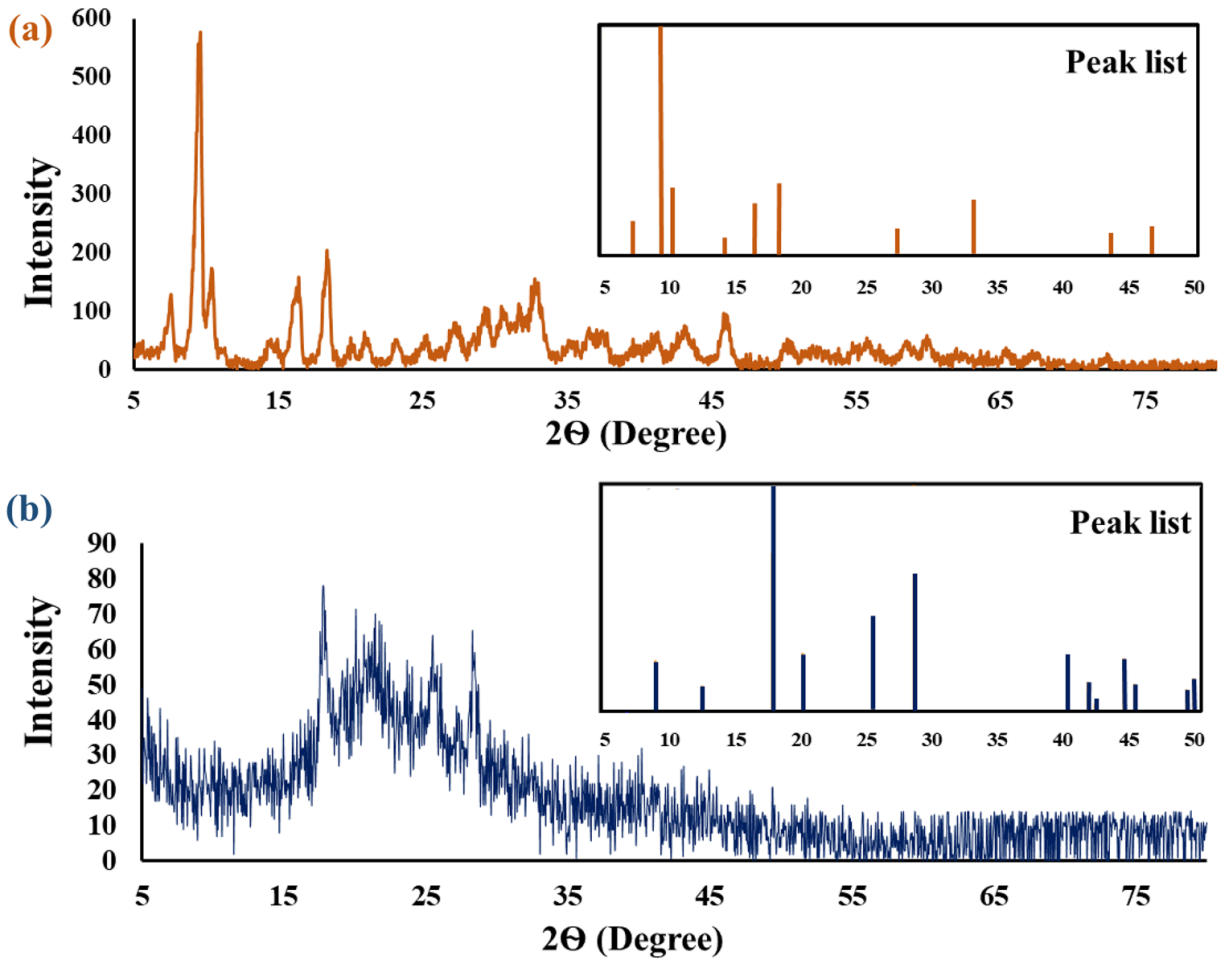
The XRD pattern of MOF-5 (**a**), and Ag-TG hydrogel/SF/MOF-5 nanobiocomposite (**b**).

Figure 5 indicates the XRD pattern of MOF-5 and prepared Ag-TG hydrogel/SF/MOF-5 nanobiocomposite. In Fig. 4a, five peaks at 2Ѳ = 7.55°, 9.60°, 14.39°, 16.44°, 18.52° were observed, which can be attributed to MOF-5, with the most prominent peak at 2Ѳ = 9.60°^52–54^. In Fig. 4b, along with the peaks corresponding to MOF-5 (2Ѳ = 9.04°, 17.84°), a broad peak related to the polymers (Ag, TG, and SF) was apparent at 2Ѳ = 19°–25°. This suggests that the nanobiocomposite possesses a combination of both crystalline MOF-5 and amorphous polymer components^55,56^.

### TGA

**Figure 6:**
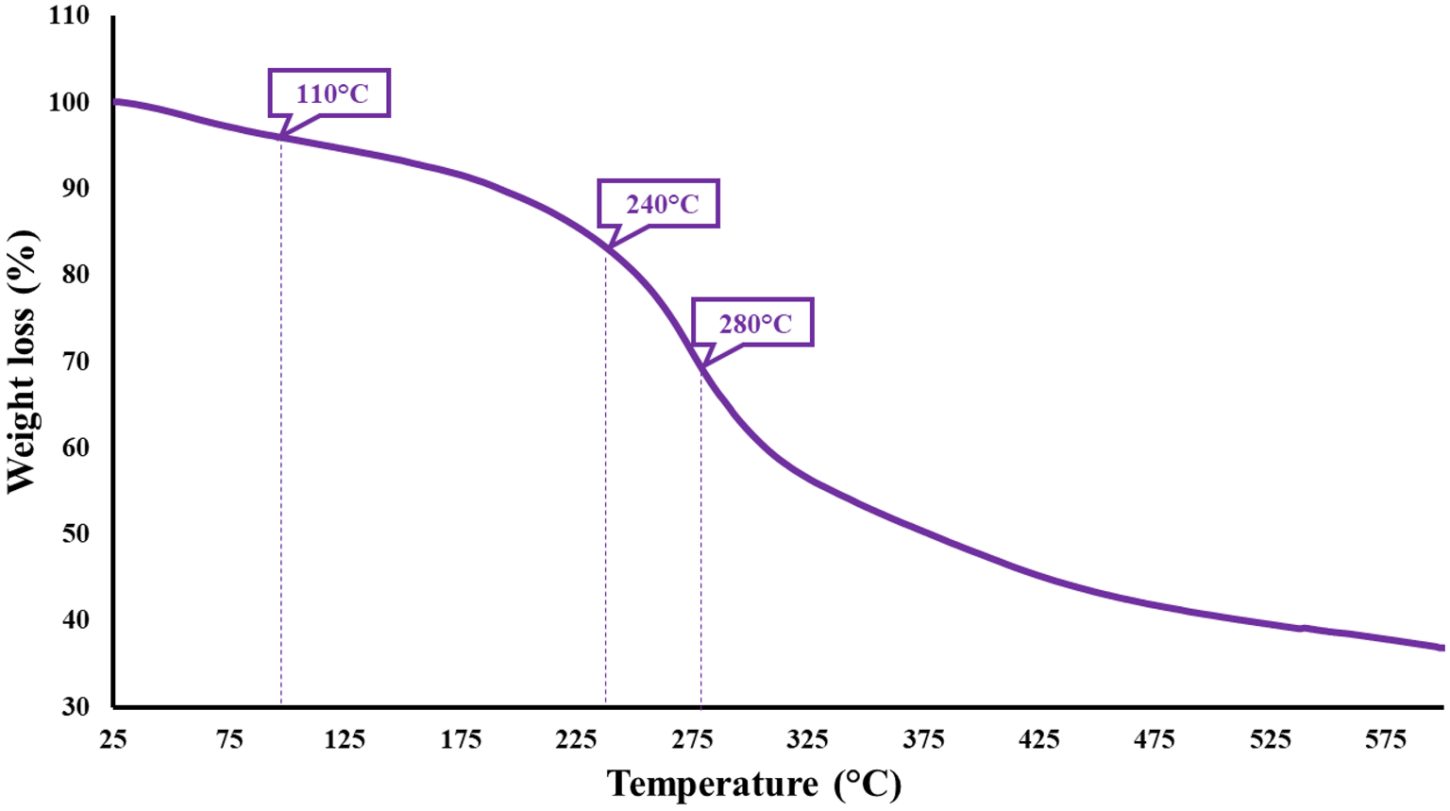
The TGA curve of Ag-TG hydrogel/SF/MOF-5 nanobiocomposite.

The TGA analysis was carried out over a temperature range of 25 °C to 600 °C, with a heating rate of 10 °C/min, using a sample weighing precisely 4.1637 mg. The obtained thermogram exhibited two significant weight loss in two distinct temperature ranges. The first weight loss, observed in the temperature range of 25–110 °C, can be attributed to the evaporation of volatile molecules and dehydration of the polymers. The second weight loss, occurring in the temperature range of 200–600 °C, can be attributed to the degradation of TG, Ag, and SF. These polymers were degraded into lower-weight molecules, including CO, CO_2_, CH_4_, NO_2_, and other volatile compounds. It is worth noting that the degradation of TG took place at a lower temperature (240 °C) compared to the degradation temperature of Ag and SF (280 °C) (Fig. 6)^57–59^.

### Cell lines proliferation assay

**Figure 7:**
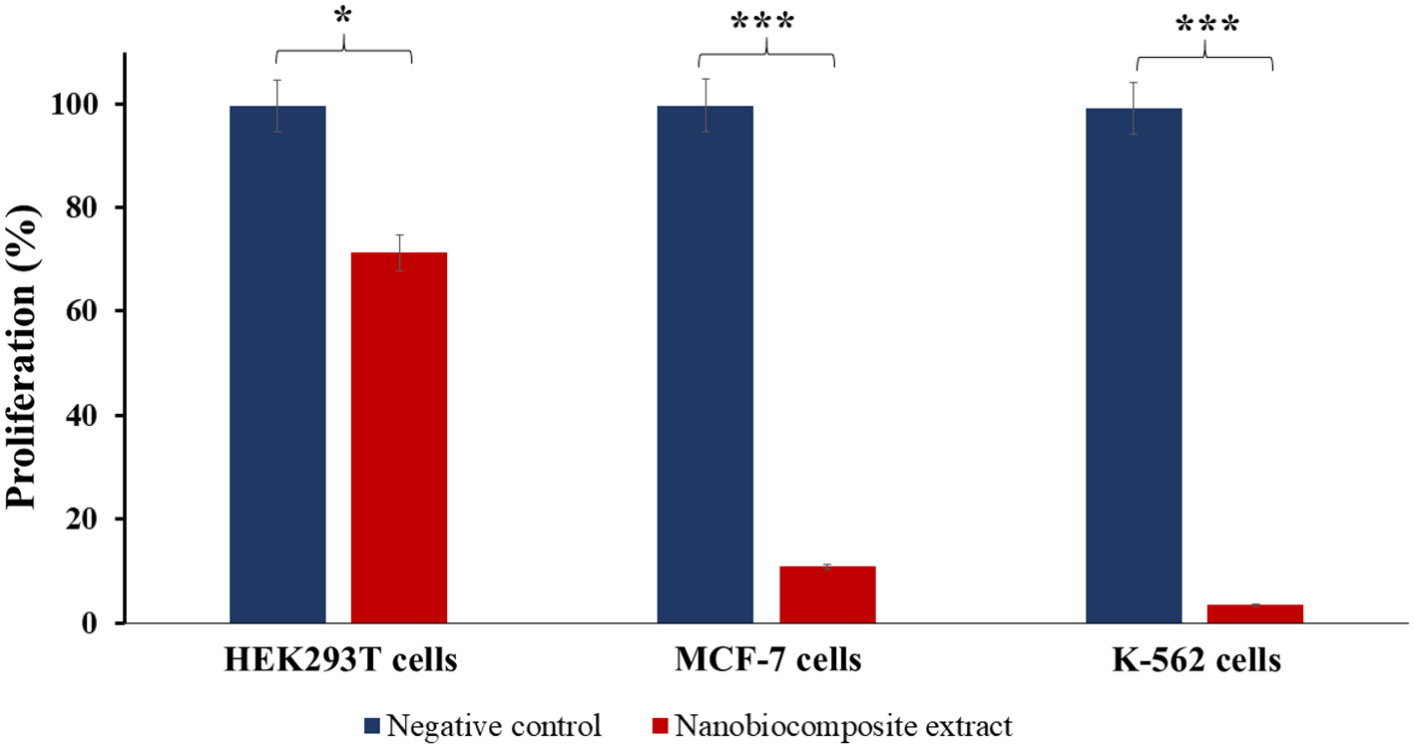
Proliferation assay of treated HEK293T, MCF-7 and K-562 cells. PBS treated (negative control) Vs. Ag-TG/SF/MOF-5 nanobiocomposite extract treated cells after 48 h (*insignificant, P ≥ 0.05 and ***very significant, P ≤ 0.001) (n = 3).

Figure 7 shows the difference between the proliferation of the treated cell lines and the control groups. The results are the average values obtained from three independent experiments.

As observed in Fig. 7, the proliferation percentage of MCF-7 and K-562 cells treated with the Ag-TG/SF/MOF-5 nanobiocomposite decreased by more than 90%, with values of 10.8% and 3.38%, respectively. These findings indicate the potential anticancer effects of the nanobiocomposite on the MCF-7 and K-562 cancer cell lines. The results demonstrated significant statistical differences between the negative control and experimental groups, where the former exhibited notably higher values (P ≤ 0.001, very significant compared to the negative control group). In contrast, the proliferation rate of HEK293T cells, a healthy cell line, was not significantly affected by the nanobiocomposite extract (P ≥ 0.05, indicating insignificance compared to the negative control group). The proliferation rate of HEK293T cells decreased by less than 30%, with a value of 71.22%. These results suggest that the Ag-TG/SF/MOF-5 nanobiocomposite is not toxic to this healthy cell line.

**Figure 8:**
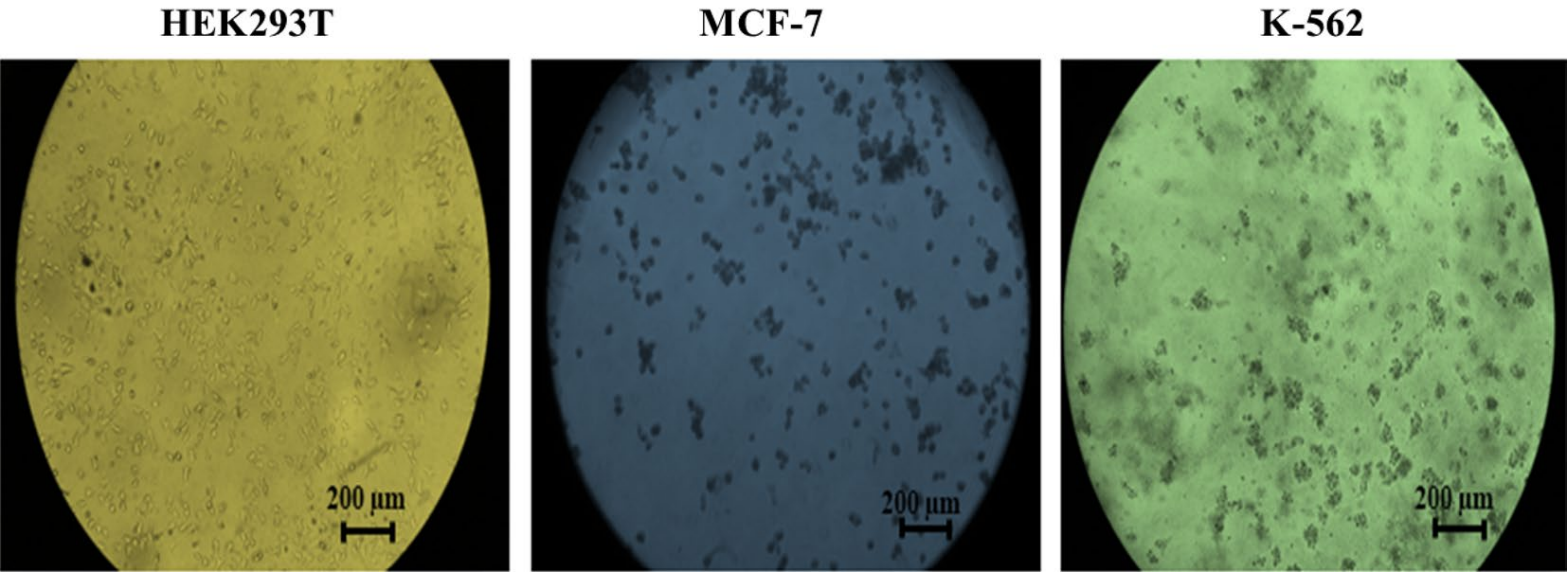
The morphological changes of Ag-TG/SF/MOF-5 nanobiocomposite extract-treated cell lines using an inverted microscope.

Figure 8 shows the morphologic differences of cells using an inverted microscope. As observed, the treated MCF-7 and K-562 cells exhibited significant morphological changes, altering their original structure. Additionally, a considerable number of these cells were lost in the medium. These cells appeared as stuck-together pieces in certain areas, indicating cell death. At the same time, no significant changes were observed in HEK293T cells after treatment. These cells did not adhere to each other and maintained their original structure. They were kept in large numbers and separately.

### Blood compatibility

The measured hemolysis of the negative control group was 1.27%, while the Ag-TG/SF/MOF-5 nanobiocomposite extract-treated RBCs exhibited a hemolysis rate of 19.88%. These results indicate a significant lytic effect of the nanobiocomposite extract (P ≤ 0.05). In comparison, the positive control, Triton X-100, demonstrated considerable hemolysis.

**Figure 9:**
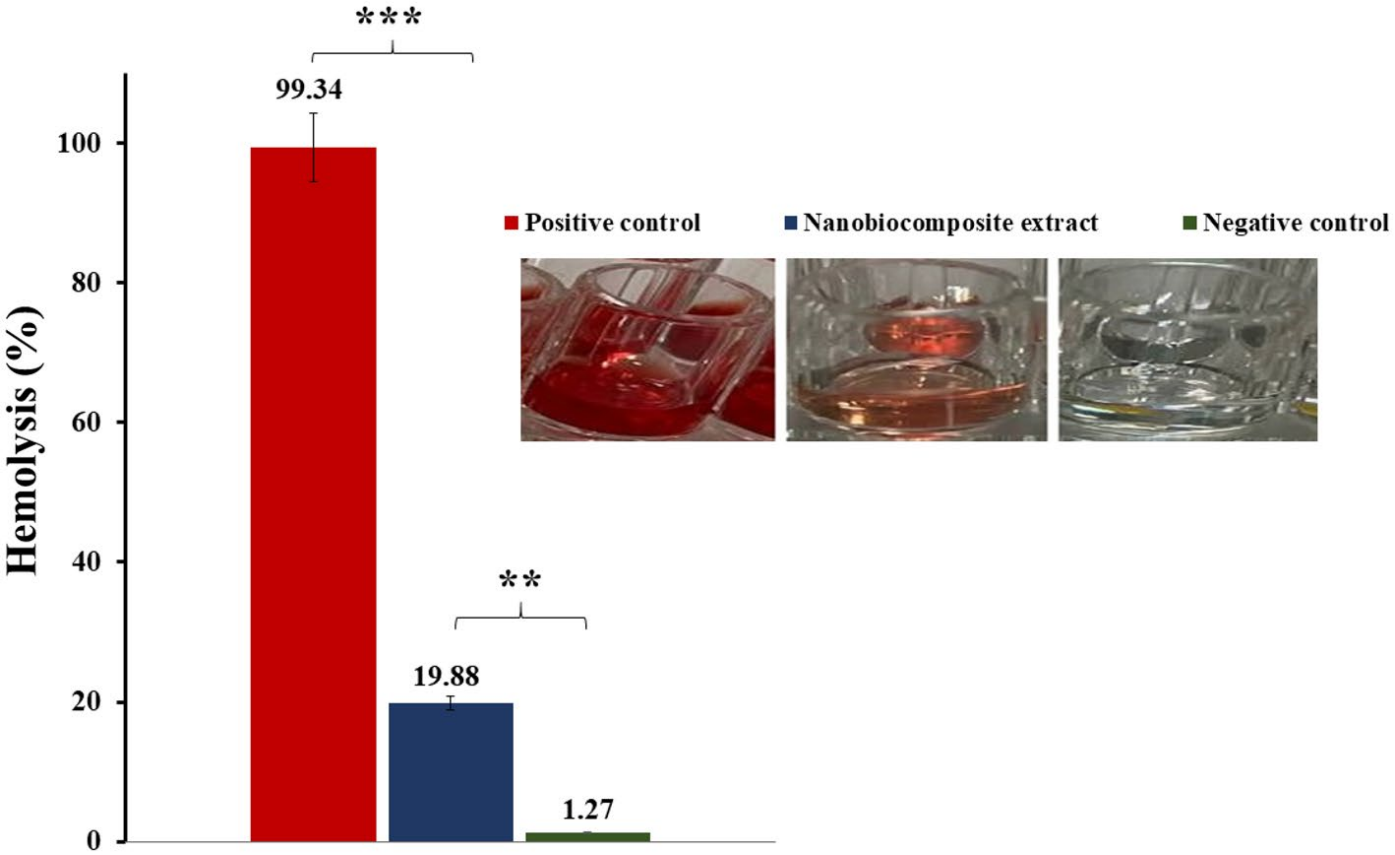
The hemolysis percentage of the Ag-TG/SF/MOF-5 nanobiocomposite and negative control-treated RBCs is presented alongside corresponding 96-well plate images (**significant, P ≤ 0.05 and ***very significant, P ≤ 0.001) (n = 3).

Figure 9 displays the hemolysis percentage and the appearance of the treated 96-well plate. It is important to note that the results presented in this report are the average outcomes from three distinct and separate trials.

### Bacteria growth inhibition

**Figure 10:**
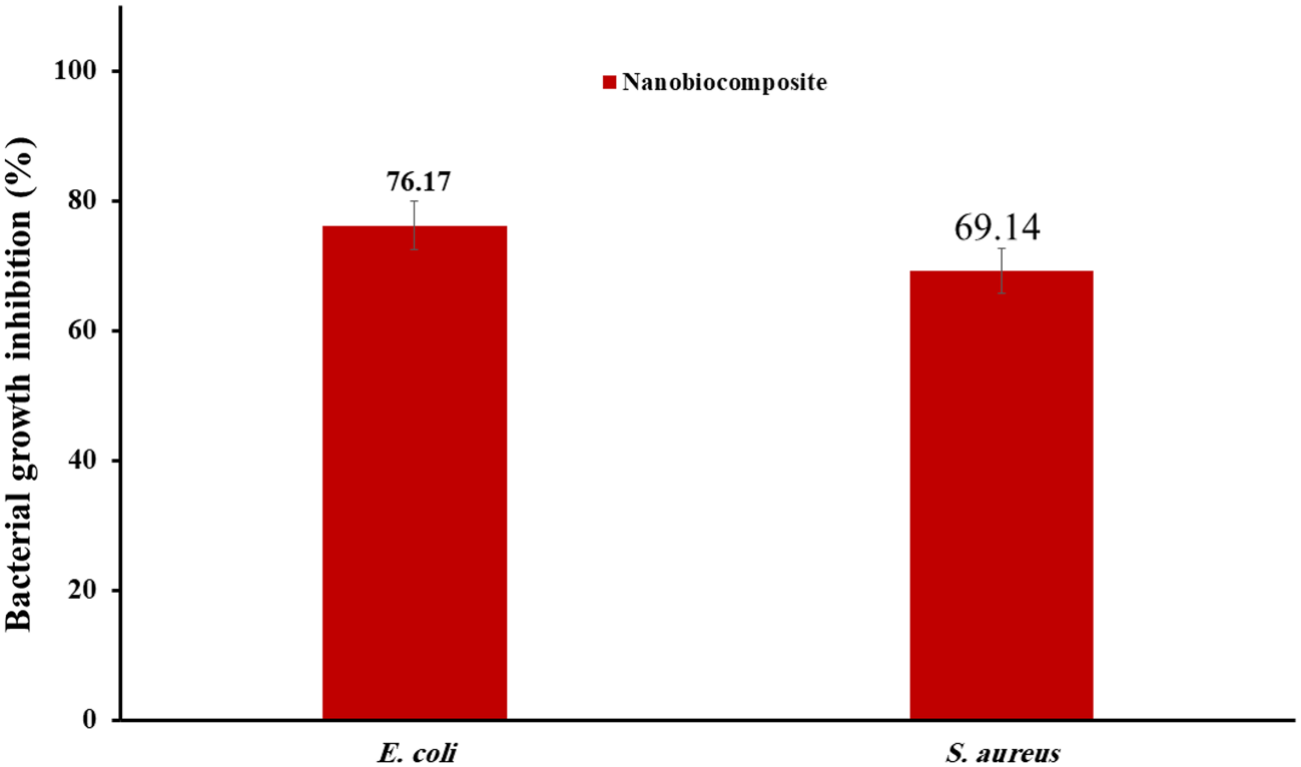
Percentage inhibition of bacterial growth (n = 3). These graphs show the ability of Ag-TG/SF/MOF-5 nanobiocomposite to prevent the growth of *E. coli* and *S. aureus* and confirm its antibacterial effects.

The Ag-TG/SF/MOF-5 nanobiocomposite exhibited an antibacterial efficacy of 76.17% against *E. coli* and 69.14% against *S. aureus* bacteria strains (Fig. 10). These results highlight the potent antibacterial activity of the nanobiocomposite. It is important to note that the reported results represent the mean of three independent experiments.

The proposed mechanism for annihilation of the bacteria involves using of Ag-TG hydrogel/SF, which can disrupt the bacterial cell membrane and cause depolarization, resulting in bacterial cell death. Furthermore, the MOFs in the nanobiocomposite can release metal ions that interfere with the bacteria's DNA, disrupt their enzyme activity, and induce oxidative stress.

## Conclusions

In this study, a novel Ag-TG hydrogel/SF/MOF-5 nanobiocomposite was synthesized using a facial approach. To check this nanobiocomposite's effect on the living cells, the proliferation of the HEK293T, MCF-7, and K-562 cells was investigated using Trypan blue exclusion method. The results show that the nanobiocomposite is biocompatible against normal cells; however, it exhibits anticancer activity against cancer cells. Moreover, the lytic effect of the nanobiocomposite was assessed by evaluating the percentage of RBC hemolysis. The antibacterial property was examined with photometric antibacterial tests against *E. coli* and *S. aureus* strains. This nanobiocomposite prevented the growth of *E. coli* and *S. aureus* (76.08% and 69.19%) with high efficiency. According to biological assays, the natural-based Ag-TG hydrogel/SF/MOF-5 nanobiocomposite offers several advantages in biological applications. It is biocompatible, reducing the risk of adverse reactions or inflammation. It is also less toxic than synthetic materials, making them safer for medical use. Additionally, the natural biomacromolecules in the nanobiocomposite are renewable, sustainable, and exhibit enhanced biodegradability without leaving harmful residues. The porosity of the nanobiocomposite is crucial in biomedical applications like wound healing. Its porous structure allows for better oxygen and nutrient exchange, supports cell attachment and proliferation, facilitates fluid absorption and retention, and enables controlled release of bioactive agents. The incorporation of MOF-5 into the Ag-TG hydrogel/SF biocomposite, enhances the antibacterial activity of the nanobiocomposite to provide additional therapeutic functionalities, such as preventing infections, and promoting wound healing. Henceforth, future research should focus on conducting more studies to explore the potential of the Ag-TG hydrogel/SF/MOF-5 nanobiocomposite in biomedical applications, particularly in wound healing and cancer therapy.

## Data Availability

All data generated or analyzed during this study are included in this published article.
